# Deletion of b1/b3 shows risk for expanse of Yq microdeletion in male offspring: Case report of novel Y chromosome variations

**DOI:** 10.1097/MD.0000000000022124

**Published:** 2020-09-11

**Authors:** Xiangyin Liu, Hongguo Zhang, Yang Yu, Jia Fei, Yuting Jiang, Ruizhi Liu, Ruixue Wang, Guirong Zhang

**Affiliations:** a Center for Reproductive Medicine, Center of Prenatal Diagnosis, First Hospital, Jilin University, Changchun; b Peking Medriv Academy of Genetics and Reproduction, Peking, China.

**Keywords:** infertile men, next generation sequencing (ngs) method, partial azfc deletions, spermatogenic failure, Y chromosome microdeletions

## Abstract

**Rationale:**

This study aimed to report 1 family case with novel Y chromosome structural variations by an established next-generation sequencing (NGS) method using unique STSs.

**Patient concerns:**

The case studied was from a family with a father and son (the proband). G-band staining was used for karyotype analysis. Y chromosome microdeletions were detected by sequence-tagged site (STS)-PCR analysis and a new NGS screening strategy.

**Diagnoses:**

Semen analysis showed that the proband was azoospermic. The patient had an abnormal karyotype (45,X[48%]/46,XY[52%]). His father exhibited a normal karyotype. STS-PCR analysis showed that the proband had a deletion of the AZFb+c region, and his father had no deletion of STS markers examined. The sequencing method revealed that the patient had DNA sequence deletions from nt 20099846 to nt 28365090 (8.3 Mb), including the region from yel4 to the Yq terminal, and his father exhibited a deletion of b1/b3 and duplication of gr/gr.

**Interventions:**

The proband was advised to undergo genetic counseling, and consider the use of sperm from a sperm bank or adoption to become a father.

**Outcomes:**

The proband was azoospermic. AZFc partial deletions may produce a potential risk for large AZFb+c deletions or abnormal karyotypes causing spermatogenic failure in men.

**Lessons:**

The NGS method can be considered a clinical diagnostic tool to detect Y chromosome microdeletions. The partial AZFc deletions and/or duplications can be a risk of extensive deletions in offspring.

## 1 Introduction

Y chromosome microdeletions are a genetic etiology of spermatogenic failure in men with infertility, with a prevalence of 5% to 10% in oligozoospermic men and 10% to 15% in azoospermic men. It has been extensively reported that microdeletions involve the azoospermia factor (AZF) regions. These deletions are mostly de novo genetic events,^[1]^ with AZFc or AZFb+c region deletions the most frequent. The detection of AZF deletions is currently recommended for patients with oligospermia and azoospermia. Apart from the diagnostic value, detection of Y chromosome microdeletions provides prognostic information for the use of testicular sperm extraction (TESE) for the treatment of infertile men. The deletions of complete AZFa, AZFb or larger regions are usually associated with Sertoli cell-only syndrome and spermatogenic arrest. Therefore, the presence of such deletions often indicates a negative factor for TESE, and these men are discouraged from going through this invasive procedure.^[2]^ Although studies have detected mature sperm in men with AZFb/b-c deletions,^[3]^ it was recommended that these patients do not receive TESE or microdissection-TESE (m-TESE) if their testicular volume was significantly smaller than normal.

Partial deletions and duplications that occur around the AZFc region have been reported. Yet, the genotypical and phenotypical characterization of partial AZFc microdeletions (like as gr/gr, b1/b3, and b2/b3) or duplications has been limited because of the restricted numbers of identified cases.^[4,5]^ Currently, screening for AZF deletions in infertile male, especially before performing assisted reproductive treatment, is usually done by polymerase chain reaction (PCR) of specific sequence tagged site (STS) markers in AZF regions. However, new structural variations, such as duplications and novel deletions, may be undetectable using these markers with routine PCR. Methods like multiplex quantitative fluorescent (QF)-PCR^[6]^ and microarray technology^[7]^ may provide a strategy to uncover other uncommon structural variations. But for clinical screening, a more comprehensive, high-resolution analysis of the Y chromosome is required. As the development of next-generation sequencing (NGS) and the associated costs decrease, such technology offers the ability to determine the whole Y chromosome sequence with high sensitivity and specificity. Thus, we have developed a new screening strategy using unique STSs and NGS technology (new NGS method) to detect known Y chromosome microdeletions and uncover the potential risk of expanded structural variations.

## 2 Case report

The proband was a 28-year-old males who presented with primary infertility for 1 year at the Center for Reproductive Medicine. A physical examination revealed that left and right testicular volumes were both 12 ml, and testis texture was normal. The patient completed a questionnaire, designed to obtain information on smoking habits, alcohol intake, profession, medication history, injuries and familial conditions, and nothing abnormal was found. He was born when his father and mother (not a consanguineous marriage) were 20 and 19 years old, respectively. The study was approved by the Ethics Committee of the First Hospital of Jilin University. Informed written consent was obtained from the patient and his father for publication of this case report and accompanying images.

## 3 Methods

### 3.1 Semen test

Ejaculated semen samples were collected by masturbation after 3 to 5 days of sexual abstinence, and semen samples were examined within 1 hour using computer assisted semen analysis (CASA) (http://www.wei-li.com/). The protocol for semen analysis was performed according to the World Health Organization (WHO) (fifth edition) (http://www.who.int/en/). The patient was diagnosed with azoospermia when no sperm was found in at least 3 ejaculates after centrifugation.

### 3.2 Karyotyping

Peripheral blood (0.5 ml) was collected in a sterile heparin anticoagulant tube and incubated in lymphocyte culture medium (Yishengjun, Guangzhou Baidi Biotech Co. Ltd, China) at 37°C for 72 hours, and then 20 mg/ml colcemid (Sigma, UK) was added for 1 hour. Lymphocytes were then harvested and processed by potassium chloride hypotonic fluid treatment, fixation in ethanol glacial acetic acid solution, trypsinization, and Giemsa banding (G-banding). Twenty metaphase cells were counted and at least 3 of these cells were analyzed.

### 3.3 Microdeletion analysis by STS-PCR

Genomic DNA was harvested from peripheral blood using a commercially available whole-blood DNA extraction kit (TIAN amp Blood DNA kit; Beijing Tiangen Biotech, Beijing, China). Specific STS markers (sY84, sY86, sY127, sY134, sY143, sY254, and sY255) spanning the entire AZF region were detected by multiplex PCR. sY14 and ZFX/Y were used as internal controls. DNA samples from a fertile male were used as positive controls. For deletion confirmation, PCR analysis was performed at least twice.

### 3.4 High throughput sequencing of the AZF region

One hundred and thirty eight locus-specific oligonucleotides for the AZF region and 10 loci for Y chromosome housekeeping genes were used as markers. These loci were collected from the human genome UCSC database (http://genome.ucsc.edu/). Specific experimental details of this sequencing method were described previously.^[8]^

## 4 Results

**Figure 1:**
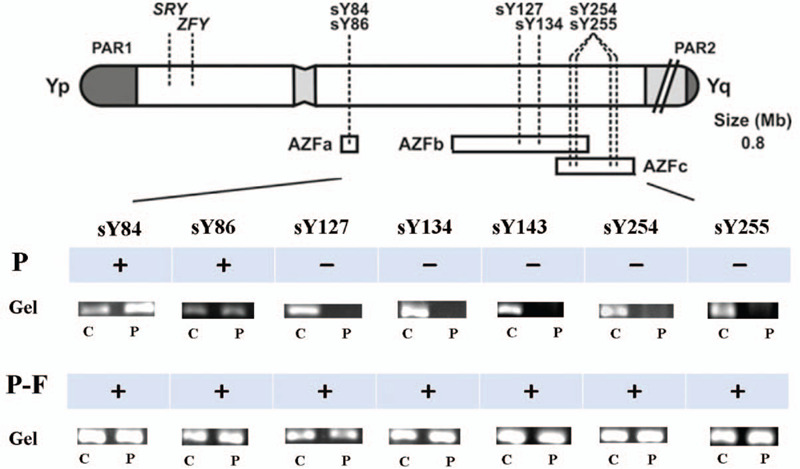
STS-PCR results on Y chromosome microdeletions, Note: P means the proband. P-F means the probands father. + means presence of STSs. —means absence of STSs. In gel electrophoresis figures, P represents the patient or his father and C represents positive control.

**Figure 2:**
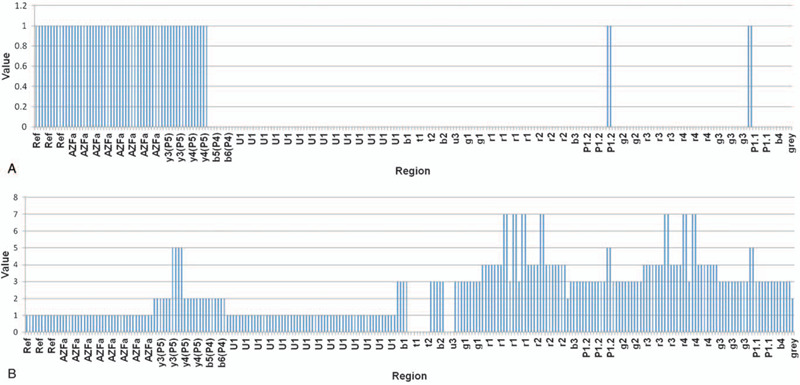
The sequencing results of the patient and his father, Note: A: Copy number of probe signals of deletions on AZFb+c (yel4 to Yq terminal) in the proband. B: Copy number of probe signals showing deletions of b1/b3 combined with duplications of gr/gr in the probands father.

The semen analysis showed that the proband exhibited azoospermia. Cytogenetic analysis showed that the proband had an abnormal karyotype (45,X[48%]/46,XY[52%]). His wife and his father both had normal karyotypes. STS-PCR analysis showed that the specific STS markers were deleted in the proband, including sY127, sY134, sY143, sY254 and sY255, indicating deletions of the AZFb+c region (Fig. 1). The STS markers sY84 and sY86 were present in the AZFa region. However, the father had no deletions of these STS markers (Fig. 1). The semiconductor sequencing method was performed for high-resolution detection of AZF microdeletions in the proband and his father. The patient had a DNA sequence deletion from nt 20099846 to nt 28365090 (approximately 8.3 Mb), which included the region from yel4 to the Yq terminal (Fig. 2A). His father exhibited the b1/b3 deletion combined with a duplication of gr/gr. Sequencing revealed the father had a 1.6 Mb deletion (24164717–25665550) and 1.6 Mb duplication (24943305–26577055) in long arm of Y chromosome (Fig. 2B).

Genetic counseling was provided to the patient. Deletions of the AZFb+c region explained the azoospermia. Therefore, TESE and related assisted reproductive techniques were not recommended. The patient was also informed about the possibility of potential male offspring having the same subfertility problem if intracytoplasmic sperm injection (ICSI) treatment was selected, as well as the consideration of other options, such as donor insemination or adoption.

## 5 Discussion

Y chromosome microdeletions have provided an important clinical target to improve the diagnostic work-up of male infertility. Currently, screening for AZF deletions is usually done by PCR analysis. But Y chromosome partial deletions or duplications and novel deletions may be undetectable using routine PCR. A high throughput and high-resolution analysis of the Y chromosome is needed for such chromosomal alterations. The complex structure of the AZF region poses challenges to sequencing this region and for the characterization of microdeletions. The NGS technology offers the ability to depict structural variations in the whole Y chromosome. In the current paper, we developed a new NGS screening strategy using unique STSs of the Y chromosome that provides the potential to detect known and novel structural variations.

In our report, the STS-PCR analysis of the proband showed deletions of the AZFb+c region, which was supported by data from the NGS method (Fig. 2A). The microdeletion spanned approximately 8.3 Mb, which included the region from yel4 to the Yq terminal, consistent with the reported literature.^[9]^ Such deletions are always associated with azoospermia, with sperm rarely observed in testicular tissue or ejaculate of these men. To date, there has been 1 published report of a pregnancy achieved via ICSI using sperm from a man with a complete AZFb+c deletion.^[10]^ Other studies showed that it was possible to sporadically detect spermatozoa in these men,^[11,12]^ indicating that the meiotic process was not completely prevented. However, during genetic counselling of the present patient, ICSI after TESE or m-TESE were not firstly recommended and other options, such as donor insemination or adoption should be considered.

Owing to its unique structure, the AZFc region is one of the most polymorphic regions in the human genome, and is particularly susceptible to non-allelic homologous recombination events. Recently, partial AZFc deletions or duplications have been described that alter the number of copies of the AZFc genes. The AZFc partial deletions, specifically gr/gr, b2/b3 and b1/b3, were associated with reduced fertility in some but not all populations.^[13]^ The functional of AZFc partial duplications remains controversial. In the present study, the NGS method revealed that the probands father had a deletion of b1/b3 simultaneously combined with a duplication of gr/gr. Despite these rearrangements, the father was fertile (producing the proband). Different studies on the clinical fertility consequences of partial AZFc deletions and/or duplications have reached different conclusions, or found it difficult to define a specific pattern of alteration associated with either a “neutral” or “pathogenic” effect. Kumari et al reported that the incidence rate of STS deletions and copy number variations had statistically significant in infertile males and normal fertile males.^[14]^ Other studies suggested that b1/b3 rearrangements have no influence on male fertility.^[15]^ However, it was found that b1/b3 partial deletions could be risk factors for male infertility (Table 1). It was reported that partial b1/b3 deletions in these men may be transmitted to their male offspring and lead to an increased risk of complete AZFc deletion in the next generation.^[25,26]^

**Table 1:**
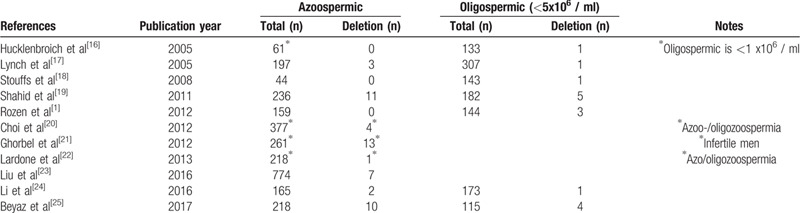
Prevalences of b1/b3 deletions among infertile men with severe spermatogenic failure.

Certain Y chromosome microduplications were thought to be common polymorphisms not associated with spermatogenesis disorders or male infertility. A Taiwanese study reported that 2 men with normal spermatogenesis had the entire AZFc region duplicated.^[13]^ However, partial AZFc duplications in a segment spanning 1.6 to 1.8 Mb were observed to be associated with spermatogenic failure in a Han Chinese population.^[27]^ Another similar study demonstrated a significantly higher prevalence of partial AZFc duplications in infertile patients than in normal fertile males.^[28]^ However, these findings have not been validated in other populations. If men with AZFc duplications were fertile, the offspring would inherit these mutated structures. Research suggested that an excess of AZFc segments may provide an additional risk for large AZFb+c deletions during the multi-stage division process of germ cells.^[29]^ Thus, partial deletions of b1/b3 or partial duplications gr/gr may ultimately disrupt the process of spermatogenesis. It is recommended that such male-factor infertile couples perform DNA-based diagnosis to assist genetic counseling and guidance for the utilization of assisted reproductive technologies.

The current proband was also found to have an abnormal karyotype, 45,X/46,XY. It remains unresolved whether this karyotypic abnormality or the Y chromosome microdeletions was the primary cause of spermatogenic impairment in this patient. It was previously reported that a high frequency of the mosaic karyotype 45,X/46,XY was found in the men with AZFb+c deletions.^[12]^ It was also reported that AZFb/c deletions may lead to an increased risk of chromosomal abnormalities, including the loss of the whole Y chromosome during embryonic development^[30]^; possibly explaining the sex chromosome mosaicism (frequently 45,X/46,XY) in patients with Y chromosome microdeletions. Therefore, in the current report, microdeletions in the Y chromosome of the probands father may have led to the chromosomal instability (rearrangements and/or Y chromosome microdeletions) in his offspring.

In conclusion, the partial AZFc deletions and/or duplications can potentially influence spermatogenic function of the carrier. These males should be informed that the deletions or duplications will be transmitted to their sons, or lead to more extensive deletions in the next generation. We have demonstrated that specific STSs combined with the NGS technique provides a suitable technique for the identification of different microdeletions on the Y chromosome. This strategy can be considered a high-resolution alternative clinical diagnostic tool for male infertility.

## Acknowledgments

We appreciate the patient and family members for their participation in this study.

## Author contributions

Ruixue Wang and Guirong Zhang contributed to the design of the article. Xiangyin Liu and Hongguo Zhang contributed to the analysis of data and wrote the manuscript. Yang Yu, Jia Fei, and Yuting Jiang contributed to the collection of data. Ruizhi Liu was the research advisor.
